# Safety and tolerability of high-dose ulinastatin after 2-hour intravenous infusion in adult healthy Chinese volunteers: A randomized, double-blind, placebo-controlled, ascending-dose study

**DOI:** 10.1371/journal.pone.0177425

**Published:** 2017-05-11

**Authors:** Qian Chen, Chaoying Hu, Ye Liu, Yun Liu, Wei Wang, Hongchao Zheng, Lianchen Rong, Jingying Jia, Shixuan Sun, Chen Yu, Yan Mei Liu

**Affiliations:** 1 Central Laboratory, Shanghai Xuhui Central Hospital & Zhongshan-Xuhui Hospital, Fudan University, Shanghai, China; 2 Department of Emergency, Shanghai Xuhui Central Hospital & Zhongshan-Xuhui Hospital, Fudan University, Shanghai, China; 3 Department of Cardiology, Shanghai Xuhui Central Hospital& Zhongshan-Xuhui Hospital, Fudan University, Shanghai, China; 4 Clinical Development Department, Techpool Bio-pharma Company, Limited, Shanghai, China; Emory University Winship Cancer Institute, UNITED STATES

## Abstract

Ulinastatin, is a broad-spectrum protease inhibitor purified from human urine, inhibits endogenous proteases such as trypsin, *α*-chymotrypsin, hyaluronidase, and plasmin. It is widely being used at increasingly higher doses for the treatment of acute or chronic pancreatitis, severe infection, and acute organ failure. We aimed to evaluate the safety and tolerability of high-dose ulinastatin in healthy volunteers in our single center, randomized, double-blind, placebo-controlled, single-dose escalation study. Fifty-one healthy Chinese subjects were enrolled in 9 dose cohorts (3×10^5^ U, 6×10^5^ U, 12×10^5^ U, 20×10^5^ U, 30×10^5^ U, 45×10^5^ U, 60×10^5^ U, 70×10^5^ U, or 80×10^5^ U of ulinastatin) and randomized to UTI or matching placebo (n = 1). Each dose cohort was composed of 3–7 subjects. All subjects were required to have 2 h of intravenous infusion. Safety and tolerability were assessed throughout the study via monitoring of vital signs, physical examinations, clinical laboratory tests, 12-lead electrocardiograms, and interviews with the subjects about adverse events. Fifty-one subjects (35 men and 16 women) completed the study. A total of 13 AEs were reported by 10 subjects: 11 adverse events in the ulinastatin groups and 2 adverse events in the placebo group. Twelve of the adverse events were possibly related to the study drug. The most common drug-related adverse events included dizziness, pain at injection site, and a decrease in white blood cell count. All adverse events were of mild severity; none were serious. In conclusion, 2 hours of intravenous infusion of ulinastatin (3×10^5^ to 80×10^5^ U) was well tolerated by healthy Chinese subjects.

## Introduction

Ulinastatin (UTI) is a broad-spectrum protease inhibitor, isolated and purified from human urine that inhibits endogenous proteases including trypsin, *α*- chymotrypsin, hyaluronidase and plasmin [1, 2]. Although the maximum recommended daily dose of UTI is 3×10^5^ U (listed in the package insert), the doses required to achieve therapeutic concentrations for severe acute diseases, such as septic shock and circulatory failure, are much higher. Physicians faced with shock, lung injury, burns, and other clinical emergencies often administer UTI at higher than recommended doses—understandable, considering the mild and controllable nature of expected adverse reactions. Indeed, the number of reports of patients receiving high doses of UTI has been increasing [3–6].

Because the current dosing recommendations do not reflect actual clinical practice, further research is needed to find the upper limit of safety. In mice and rat, the lethal dose 50% (LD_50_; an indicator of a substance’s acute toxicity) of UTI is greater than 3 × 10^6^ U/kg (equivalent to more than 14.4 or 28.8 million units/60kg in humans) [7]. Toxicology studies were conducted by Techpool Bio-pharma Company as application materials required by CFDA. The maximum tolerated dose (MTD) of UTI administered intravenously and intraperitoneally in mice were greater than 3.75 × 106 U/kg (equivalent to more than 18 million units/60kg in humans). Long-term animal toxicity tests in dog have shown no toxicity at 5 × 10^5^ U/kg, which is equivalent to 16.2 million units/60kg in humans, approximately 54 times greater than current dosing guidelines. A tolerance study conducted at the First Affiliated Hospital of Anhui Medical University in 2003 showed that high-dose UTI is well tolerated by healthy subjects [8], but the dose escalation used in that study was only 3 × 10^5^ to 12 × 10^5^ U. Thus, we conducted this ascending-dose study (3×10^5^ to 80×10^5^ U) to investigate the safety and tolerability of high-dose UTI in healthy Chinese subjects.

## Materials and methods

This study was registered with the Chinese Clinical Trial Registry (registration number: ChiCTR-TRC-14005176). It was conducted at Shanghai Xuhui Central Hospital (Shanghai, China) from August to October 2014 in accordance with the principles of the Declaration of Helsinki[9], Good Clinical Practice guidelines [10] and other applicable regulatory requirements. The study protocol, protocol amendments, and informed-consent form were approved by the Independent Ethics Committee (IEC) of Shanghai Xuhui Central Hospital on 15^th^ July 2014 (Approval No. 2014–09). All subjects gave written informed consent before participation.

### Study drug and administration

Ulinastatin for injection (Tianpuluoan; 1×10^5^ U/vial; batch number: 031406113), a white or slightly yellow powder, was provided by Guangdong Techpool Bio-pharma Co., Ltd., Guangdong, China. It was diluted in 250 mL of normal saline and infused intravenously over a 2-h period.

The placebo (batch number: P120201) was provided by Guangdong Techpool Bio-pharma Co. Ltd., Guangdong, China. It contained the excipients from the pharmaceutical preparation of UTI (mannitol, sodium chloride, and phosphate buffer).

### Selection of subjects

The sample size was primarily determined by feasibility and regulatory requirements. Subjects who met the inclusion criteria but not the exclusion criteria were enrolled in the study. The inclusion criteria were as follows: (1) Chinese men or women aged 18–45 years; (2) body mass index between 19 and 28 kg/m^2^; (3) no history of cardiovascular, pulmonary, hepatic, renal, hematologic, gastrointestinal, immune, skin, endocrine, neurological, or psychiatric diseases; (4) no history of allergies; (5) agreed to use of an acceptable method of contraception (oral contraceptive, condom, estrogen vaginal ring, or double-barrier method). Exclusion criteria: (1) evidence or history of tumor within 5 years of screening; (2) positive for hepatitis B virus surface antigens or hepatitis C virus, HIV, or syphilis antibodies; (3) use of any drugs within 2 weeks prior to screening; (4) participation in any clinical trials within three months; (5) blood donation within three months; (6) clinically significant findings upon physical examination, 12-lead electrocardiograms (ECGs), vital sign measurements, and/or clinical laboratory tests; (7) drugs or alcohol abuse.

### Study design

**Fig 1:**
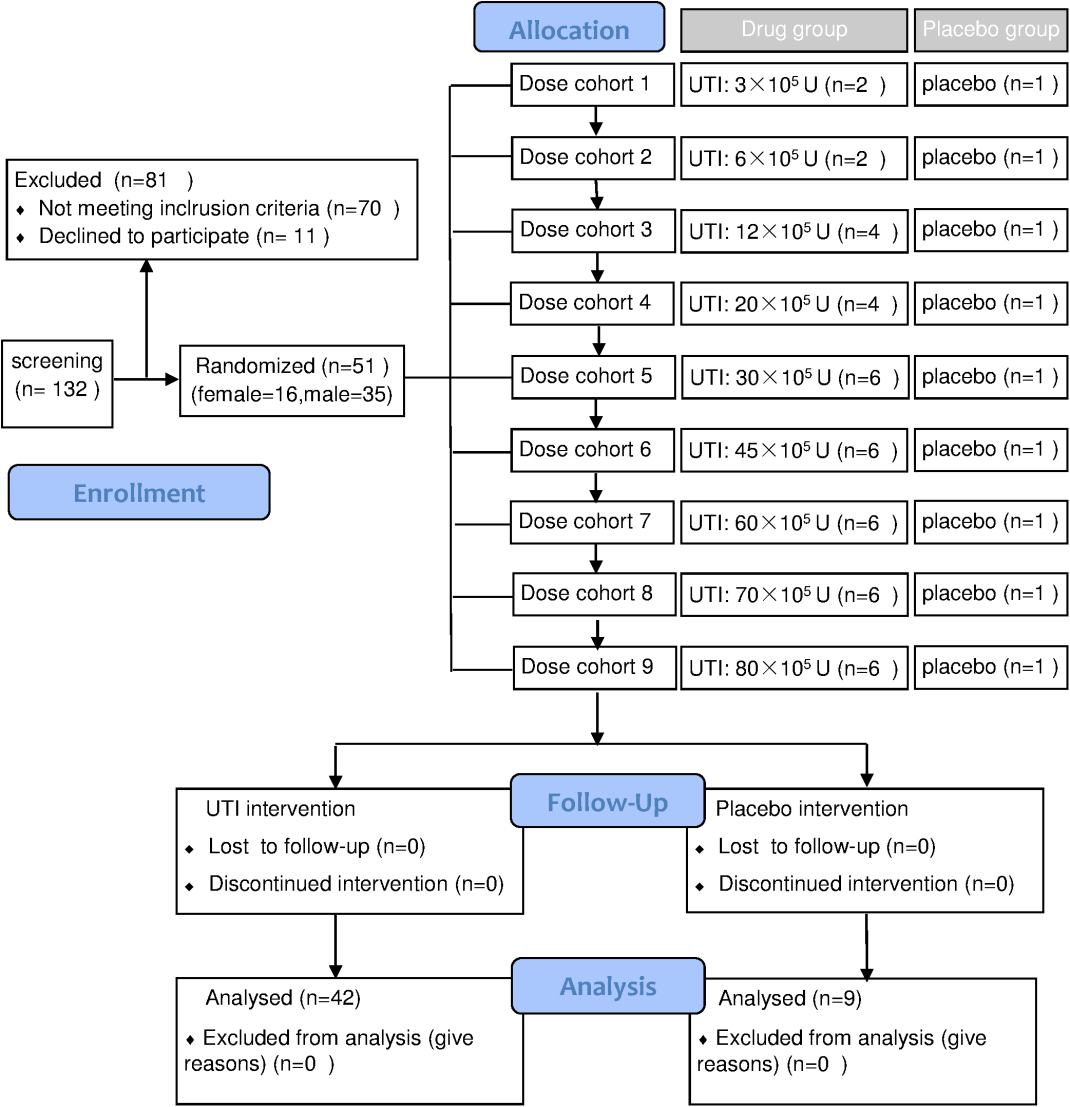
Study design and group distribution.

This was a single-center, randomized, double-blind, placebo-controlled, single-dose escalation design study that included nine dose cohorts (3×10^5^ U, 6×10^5^ U, 12×10^5^ U, 20×10^5^ U, 30×10^5^ U, 45×10^5^ U, 60×10^5^ U, 70×10^5^ U, and 80×10^5^ U of UTI). Fifty-one healthy Chinese subjects were enrolled in one of 9 dose cohorts. One random subject from each cohort received matching placebo using a table of random numbers generated by the quality assurance personnel of the clinical unit. This randomization was a block design that was concealed from all investigators in the study. Doses were administered in a serial manner proceeding from the lowest to the highest dose (Fig 1). The study was to be halted for any serious adverse reactions (determined by the Common Terminology Criteria for Adverse Events, version 4.0), or if over half of the subjects experienced adverse events of Grade 2 or greater.

Beginning 1 week before admission and continuing through the end of the study, subjects were prohibited from smoking, taking medications, engaging in strenuous exercise, and having food or beverages containing alcohol, caffeine, and/or grapefruit juice. The day before dosing (Day 0), all subjects checked into the Phase I Clinical Research Unit of Shanghai Xuhui Central hospital at about 5:00 PM and were given the same standardized dinner. On Day 1, they received an intravenous infusion of either UTI or placebo 1 h after breakfast. Vital signs were examined before dosing, and then 30 min, 1 h, and 2 h after study drug administration; In addition, a 12-lead ECG was taken 2 h after drug administration. The same standard meals were served 4 and 9 h after dosing. All subjects were confined and closely monitored for 24 h after the end of infusion. They were discharged after 24 h tolerability assessments were completed. Telephone follow-ups were conducted within 7–10 days after dosing.

### Safety assessments

Safety and tolerability were assessed through vital signs (oral temperature, heart rate, respiratory rate, and sitting blood pressure), physical examinations, clinical laboratory tests (urinalysis, hematology, and blood chemistry), 12-lead ECGs, and interviews with the subjects about adverse events at baseline (Day 0) and completion of the study (24 h post-dose).

The study protocol required that all adverse events (AEs) and serious adverse events (SAEs) that occurred throughout the study period be recorded in the source data record and on a case report form. The investigator was responsible for evaluating all AEs in terms of intensity (mild, moderate, or severe), duration, severity, outcome, and the relationship to the study drug.

### Statistical analysis

All subjects who received at least one dose of the study drug were included in the safety analyses. Those who received placebos were combined into a placebo group. Statistical analyses were performed using SAS version 9.2 software (SAS Institute Inc., Cary, NC, USA). Safety parameters were summarized descriptively. Adverse events were coded using the Medical Dictionary for Regulatory Activities. The number of subjects having treatment-emergent AEs were tabulated using primary System Organ Classes. The incidences of AEs between the UTI groups and the placebo group, and between men and women were compared using Fisher’s exact probability method.

## Results

### Demographics and baseline characteristics

A total of 51 healthy Chinese male (N = 35) and female (N = 16) volunteers were enrolled, with a mean (standard deviation, SD) age of 26.8 (5.2) years, weight of 60.9 (6.4) kg, height of 167.1 (7.7) cm, and body mass index of 21.8 (2.1) kg/m^2^. All participants completed the study as planned. The demographics and baseline characteristics of each group are shown in Table 1.

**Table 1:** Demographic and baseline characteristics of each cohort. Data are presented as mean ± SD.

Drug dose (10^5^ units)	UTI group										Placebo (n = 9)	Total (n = 51)
	3 (n = 2)	6 (n = 2)	12 (n = 4)	20 (n = 4)	30 (n = 6)	45 (n = 6)	60 (n = 6)	70 (n = 6)	80 (n = 6)	Total (n = 42)		
Male	1	1	2	2	3	4	4	4	6	27	8	35
Female	1	1	2	2	3	2	2	2	0	15	1	16
Age (year)	24.5±6.4	34.0±2.8	31.8±6.8	24.3±2.4	26.2±2.5	26.2±5.9	26.2±7.7	26.5±4.9	28.5±4.7	27.2±5.4	24.8±3.5	26.8±5.2
Height (cm):	164.8±5.2	162.2±12	161.1±3.4	165.6±5.3	161.3±10	171.2±10	170.2±8.6	164.2±4.3	171.5±5.4	166.4±8.2	170.2±3.4	167.1±7.7
Weight (kg):	53.3±2.4	60.0±8.5	58.8±3.7	62.7±9.3	56.4±4.2	64.8±3.2	61.2±6.5	60.0±7.8	63.0±6.1	60.6±6.3	62.4±7.1	60.9±6.4
BMI (kg·m^-2^)	19.7±0.4	22.8±0.4	22.7±1.2	22.8±2.2	21.8±2.3	22.2±2.2	21.1±1.1	22.2±2.8	21.5±2.6	21.9±2.0	21.5±2.4	21.8±2.1

**Abbreviations:** BMI, body mass index; SD, standard deviation.

### Safety and tolerability

No SAEs were reported and no subjects discontinued the study because of AEs. A total of 13 AEs were reported by 10 subjects (4 men and 6 women) during the study. The investigator believed that 12 AEs were possibly related to the study drug. The most common drug-related AEs were dizziness, pain at the injection site, and reduced white blood cell count. All AEs were transient and of mild severity. The details are shown in Table 2.

**Table 2:** List of adverse events and AE incidence.

AE	Placebo group	UTI group(10^5^ units)									
		3	6	12	20	30	45	60	70	80	Total
N (M/F)	9 (8/1)	2 (1/1)	2 (1/1)	4 (2/2)	4 (2/2)	6 (3/3)	6 (4/2)	6 (4/2)	6 (4/2)	6 (6/0)	42 (27/15)
AE (M/F)	2 (1/1)	1 (0/1)	0 (0/0)	1 (0/1)	1 (0/1)	1 (1/0)	2 (1/1)	0 (0/0)	2 (1/1)	0 (0/0)	8 (4/6)
Drug-related AE (M/F)	2 (1/1)	1(0/1)	0 (0/0)	1 (0/1)	1 (0/1)	0 (0/0)	2 (1/1)	0 (0/0)	2 (1/1)	0 (0/0)	7 (3/6)
Dizziness	0	0	0	1	1	0	1	0	0	0	3
Pain at injection site	0	1	0	1	0	0	0	0	0	0	2
Nausea	0	0	0	1	0	0	0	0	0	0	1
Vomiting	0	0	0	1	0	0	0	0	0	0	1
Allergic dermatitis	0	0	0	0	0	0	0	0	1	0	1
Rhinorrhea	0	0	0	0	0	1	0	0	0	0	1
White blood cell decreased	0	0	0	0	0	0	1	0	1	0	2
Blood bilirubin increased	1	0	0	0	0	0	0	0	0	0	0
Hyperuricemia	1	0	0	0	0	0	0	0	0	0	0

**Abbreviations:** M: male; F: female.

From the study groups, 11 AEs were reported by 8 subjects (8/42, 19% (95%CI: 9%-34%)), including an AE (rhinorrhea) that was unrelated to the study drug. Of the 11 AEs, 9 were symptomatic (3 dizziness, 2 pain at injection site, 1 nausea, 1 vomiting, 1 allergic dermatitis, and 1 rhinorrhea), and 2 were abnormal laboratory results (reduced white blood cell count). There were no unexpected AEs.

From the placebo group, 2 AEs were reported in 2 subjects (2/9, 22% (95%CI: 3%-60%)): increased blood bilirubin and hyperuricemia. Both AEs were transient and the subjects recovered within 7 days.

Using Fisher’s exact probability test, we found that the incidence of AEs did not differ between drug and placebo groups (P = 1.000), nor did it differ between males and females (P = 0.054).

## Discussion

UTI inhibits multiple proteases and the excessive release of oxygen free radicals and inflammatory mediators, and improves microcirculation [11–13]. Thus, it is widely used in clinical setting to treat acute or chronic pancreatitis, severe infection, septic shock, acute circulatory failure and multi organ failure[14–17]. However, the doses used in clinical practice often exceed the manufacturer’s recommendations, which may pose serious risks. Therefore, a phase I clinical study of UTI in a randomized, double-blind, placebo-controlled, dose-escalated trial in healthy Chinese volunteers was necessary in order to study the clinical safety and tolerability of intravenous UTI infusions.

In consideration of the actual needs of clinical treatment and the safety of healthy subjects, the highest dose was set as 8 million units, which exceeds the levels currently used in clinical practice. However, 8 million units may safe according to animal toxicology studies. The study results suggested UTI was generally well tolerated by our volunteers. All AEs were of mild intensity, with the subjects recovering spontaneously within 12 days. There were no SAEs and unexpected AEs, and the most frequently reported AEs were consistent with those reported in clinical practice (dizziness, pain at the injection site, and reduced white blood cell count). These results suggest that the dosing guidelines in the package inserts should be revised. The current maximum clinical therapeutic dose of 5 million units per day is safe, and it could potentially be increased if clinically necessary. Furthermore, the results suggest that the drug strengths available on the market are not high enough to meet the needs of high-dose administration. Therefore, we propose that the strength of UTI preparations be appropriately increased. It is better to conduct the multiple doses study to confirm it.

Although this study failed to reveal the maximum tolerated dose of UTI in healthy subjects, it confirmed that healthy subjects can tolerate a single dose of 8 million units. The frequency of AEs did not differ between the UTI and placebo groups, and between men and women. However, our study was limited by the small number of female subjects enrolled. Further studies should be conducted to investigate the effects of gender differences. Nevertheless, the information we present here lays a foundation for future follow-up studies of ulinastatin pharmacokinetics.

## Conclusions

We found that intravenous infusion of 3×10^5^ to 80×10^5^ U of UTI over 2 h was well tolerated by healthy Chinese subjects.

## Supporting information

S1 File CONSORT checklist. (DOC)

S2 File Study protocol (English version). (PDF)

S3 File Study protocol (Chinese version). (PDF)
