# Supplementary material for: Safety and tolerability of high-dose ulinastatin after 2-hour intravenous infusion in adult healthy Chinese volunteers: A randomized, double-blind, placebo-controlled, ascending-dose study
Source: PLoS One. 2017 May 11;12(5):e0177425. doi: 10.1371/journal.pone.0177425 (PMC5426756; doi:10.1371/journal.pone.0177425)
Supplement: S2 File — (PDF) [file pone.0177425.s002.pdf]

A randomized, double blind, placebo control, single dose escalation study for evaluating safety and tolerance of ulinastatin in healthy subjects (Protocol number: CRC-C1406)

Study Site: Shanghai Xuhui Central Hospital

Investigator: Yu Chen

Statistical unit: Shanghai Clinical Research Center

CRO: Shanghai Clinical Research Center

statistician: Fu guangjian

Sponsor: Guangdong Techpool Bio-pharma Co., Ltd.

Contact person: Rong Lianchen

The document contains trade secrets and confidential commercial information. They can’t be disclosd without informing the sponsor and getting its permission.

Signature page

Title: A randomized, double blind, placebo control, single dose escalation study for evaluating safety and tolerance of ulinastatin in healthy subjects (Protocol number:CRC-C1406; version: V2.0. Date: 2014-06-30)

1． Sponsor

We have read and confirmed the protocol, agreed to carry out this clinical trial according to the protocol. We will conscientiously fulfill the duties of sponsor according to China's GCP regulations and are responsible for initiating, applying, organizing, financing and auditing the clinical trial. In particular, we will provide compensation for the related subjects who are injured or dead during the study, and bear the associated treatment cost and certain financial compensation.

Sponsored by: Guangdong Techpool Bio-pharma Co., Ltd.

Address: Room 3060, Libao Square, No.222, Central Huaihai Road, Shanghai Postal code: 200021

Representative of sponsor: (signature): Date of signature:

2． Investigator

I have read and confirmed the protocol, agreed to conduct the study according to the protocol. I will conscientiously perform the duties of investigator according to China's GCP regulations, participate or directly guide the clinical research. I agree to perform relevant duties in accordance with Chinese laws, the principle of Declaration of Helsinki, China's GCP and this research scheme. In addition, the scheme can only be revised after the sponsor has been informed. The revision can only be implemented after getting the consent from Ethics Committee unless the measures must be taken to protect safety, rights and interests of the subject. I will be responsible for making clinically relevant medical decisions to ensure appropriate treatment can be promptly conducted for the subject when adverse events occur during the research. Moreover, these adverse events are recorded and reported in accordance with relevant national regulations. I guarantee that the data will be truly, accurately, completely and promptly loaded into the medical records for research. I will accept supervision and inspection made by associate of clinical research or inspector dispatched by the sponsor, as well as inspection and survey by drug supervision and administration department to ensure the quality of clinical trial. I promise to keep the secrets of subject information and related matters. I have been told that if the commitment is broken, I would assume resulting legal liability. I agree to disclose my own full name and occupation to the sponsor, disclose the expenditures related to clinical research and prohibit related commercial and economic behaviors of the test.

Study Site： Shanghai Xuhui Central Hospital

Address: F/20, No.1 building, No.966, Central Huaihai Road, Shanghai Postal code: 200031

Primary investigator (signature): Date of signature:

Synopsis

Drug name: Ulinastatin for Injection	Protocal Number：CRC-C1406
Sponsor: Guangdong Techpool Bio-pharma Co., Ltd.	Indications: (1) Acute pancreatitis; (2) Chronic recurrent pancreatitis; (3) Adjuvant for rescuing acute circulatory failure.
Protocol Title: A randomized, double blind, placebo control, single dose escalation study for evaluating safety and tolerance of ulinastatin in healthy subjects	
Study design: single center, randomized, double blind, placebo controlled, dose escalation study	
Study Population: Healthy Chinese adult subjects.	
Objective:<br>Evaluate safety and tolerance of ulinastatin after single intravenous infusion in healthy subjects.	
**Drug Administration:**<br>Ulinastatin injection (100,000 units/vial) and placebo (only contain auxiliary materials); Study drug was diluted in 250 mL of normal saline and infused over a 2-hour period.	
**Evaluation Indicators:**<br>Safety and tolerance：Incidences of adverse events, including abnormalities of symptoms / signs and laboratory.	

Overall Design：

This study is a single center, randomized, double blind, placebo controlled, single dose escalation design.

The study will be carried out strictly following the principle of dose escalation: Doses will be administered in a serial manner proceeding from the lowest dose level to the highest. The dose escalation study would be terminated if standard of test termination occurs during the process of dose escalation. Although the termination standard doesn't occur when maximum dose is achieved, the test should also be terminated.

After the clinical trial for each dose group, safety analysis should be promptly implemented in order to adjust the subsequent test scheme.

The proportion of female subjects should not be less than 30%.

Dose group for administration is set in the following table:

Group No.	1	2	3	4	5	6	7	8	9
Ratio of increase	Initial dose	100%	100%	67%	50%	50%	33%	16%	14%
Dosage (10^4 units)	30	60	120	200	300	450	600	700	800

Subject (drug+placebo)	2+1		2+1	4+1	4+1	6+1	6+1	6+1	6+1	6+1

Study process:

Screening of subjects will take place 28 days before the study in accordance with the subject inclusion and exclusion criteria. Signed informed consent form must be obtained before screening.

All subjects eligible check in the Phase I Clinical Research Unit of the hospital at the day before the dosing day and received the same standardized dinner. The subjects take breakfast at about 7:30am in the dosing day morning and receive UTI or placebo with intravenous infusion at 8:30am. Vital signs were performed at pre-dose and 30minutes, 1 hour and 2 hours (after termination of the 2-hour infusion) after study drug administration ; 12-lead ECG was taken at 2 hours after drug administration. All subjects were under close medical monitoring during confinement to the clinical Unit for a 24-hour period after drug administration and were discharged after the 24-hour tolerability assessments were completed. The subjects have lunch and dinner at 11:30 and 17:30, respectively.

Telephone follow-up should be conducted within 7-10 days after dosing. Subjects were prohibited from smoking, taking medications, having food or beverages containing alcohol, caffeine, and/or grapefruit juice, and from engaging in strenuous exercise 1 week before or during the study.

Inclusion Criteria (subjects must meet following 4 inclusion criteria):

1. Healthy Chinese male or female subjects: A condition of general good health, based upon the results of medical history, physical examination, vital signs, 12-lead ECG, chest X-ray, laboratory test;
2. Age: ≥18 and ≤45;
3. Body weight: ≥45 kg, body mass index (BMI): ≥19 and ≤28 kg/m2;
4. Be able to understand and comply with scheduled visits, treatment plan, blood sample collection , and other study procedures. Subjects must voluntarily sign and date each informed consent.

Exclusion Criteria (subjects presenting with any of the following will not be included in the study):

1. More than 5 cigarettes per day before screening;
2. History of drug or alcohol abuse;
3. Evidence or history of cardiovascular, pulmonary, hepatic, renal, hematologic, gastrointestinal, immune, skin, endocrine, neurological or psychiatric deseases;
4. Evidence or history of tumor within 5 years before screening;
5. History of ECG abnormalities with clinical significance;
6. Any disorder or abnormalities that may alter the absorption, metabolism or clearance of drug;
7. Evidence or history of cardiovascular, respiratory, hepatic, renal, gastrointestinal, endocrine or neurological system deseases;
8. Abnormal laboratory test result with clinical significance;
9. Medical history or evidence with renal insufficiency that is manifested as clinical significant

abnormalities of creatinine, urea nitrogen or urine components (such as proteinuria);

10. Suffer from severe illness, severe infection or injury within two weeks before drug administration;
11. Donation or loss of 400 mL or more blood volume within three months before drug administration;
12. Allergic history of drug or food;
13. Participate in any clinical trial within three months before drug administration;
14. Use of prescription drug, non-prescription drug, Chinese herbal medicine, food supplement (including vitamins) or any drug that may interfere with safety data or has known toxic effect on major organs within 2 weeks prior to screening;
15. Pregnant or breastfeeding female. Male or female who has fertility and is reluctant to use acceptable method of contraception;
16. Positive test result for Hepatitis B surface antigen, hepatitis C virus antibody, HIV antibody or syphilis antibody;
17. Consideration by the investigator, for any reason, that the subject is an unsuitable candidate for the study.

Withdraw criteria：

1. Subjects who experience treatment related adverse events that are intolerable, and are deemed by the investigator as unsuitable to continue the study;
2. Protocol deviation (Subjects who is deemed by the investigator as non-compliant);
3. Subjects who voluntarily withdraw from the study.

Termination criteria:

1. If serious adverse reaction occur during dose evaluation, though maximum dose isn’t reached, the test should be terminated;
2. If over half of subjects experienced grade 2 or greater adverse reaction according to CTC AE 4.0 (Common Terminology Criteria for Adverse Events) at the individual dose level, the test should be terminated;
3. It’s difficult to evaluate the drug due to sever protocol deviations;
4. The sponsor requires termination due to the reasons of finance or management;
5. Regulatory agencies or Ethics Committee terminate the study due to safety concerns.

Statistical Methods:

Statistical analyses were performed using SAS version 9.2. All subjects who received at least one dose of study drug were included in the safety analyses. Demographic variables and safety parameters were summarized descriptively. Calculate the incidence of adverse events and adverse reaction.

Expected duration of study： July 2014 - December 2014
