# Supplementary material for: Safety and tolerability of high-dose ulinastatin after 2-hour intravenous infusion in adult healthy Chinese volunteers: A randomized, double-blind, placebo-controlled, ascending-dose study
Source: PLoS One. 2017 May 11;12(5):e0177425. doi: 10.1371/journal.pone.0177425 (PMC5426756; doi:10.1371/journal.pone.0177425)
Supplement: S3 File — (PDF) [file pone.0177425.s003.pdf]

一项在健康受试者中评价乌司他丁单次剂量递增的随机、双盲、安慰剂对照的安全性、耐受性研究（项目编号：CRC-C1406）

临床试验机构：上海市徐汇区中心医院药物临床试验机构

主要研究者：余琛

统计单位：上海医药临床研究中心有限公司

合同研究组织：上海医药临床研究中心有限公司

统计负责人：付广建

申办单位：广东天普生化医药股份有限公司

联系人：戎练晨

本文件包含商业秘密和机密的商业信息，未事先通知申办方并获得许可的情况下，严禁泄露。

方案同意签字页

方案名称：一项在健康受试者中评价乌司他丁单次剂量递增的随机、双盲、安慰剂对照的安全性、耐受性研究（项目编号：CRC-C1406，版本号：V2.0，版本日期：2014-06-30）

1．申办者

我方已阅读并确认此临床试验方案，同意按照本方案设计及规定开展此项目临床研究。我方将根据中国 GCP 规定认真履行申办者职责，负责发起、申请、组织、资助和监查本项临床研究，特别对临床研究中发生与研究相关的损害或死亡的受试者提供赔偿，承担相关治疗费用和一定的经济补偿。

申办单位：广东天普生化医药股份有限公司

地址：上海市淮海中路 222 号力宝广场 3606 室　　邮编：200021

申办方代表（签名）：签字日期：

2．研究者

我方已阅读并确认此临床试验方案，同意按照本方案设计及规定开展此项目临床研究。我将根据中国 GCP 规定认真履行研究者职责，亲自参加或直接指导本临床研究。我同意按照中国法律、赫尔辛基宣言的原则、中国 GCP 以及此研究方案履行相关的职责，并只有在通知申办者后才对方案进行修改，需经伦理委员会同意后才可实施，除非为保护受试者的安全、权利和利益而必须采取措施。我将负责做出与临床相关的医学决定，保证受试者在研究期间出现不良事件时及时得到适当的治疗，并按照国家有关规定要求记录和报告这些不良事件。我保证将数据真实、准确、完整、及时地载入研究病历。我将接受申办者派遣的监查员或稽查员的监查和稽查及药品监督管理部门的稽查和视察，确保临床试验的质量。我承诺保守有关受试者信息和相关事宜的秘密。我已被告知，如果违背承诺，我将承担由此导致的法律责任。我同意向申办方公开自己的完整姓名、职业，同意应要求公开与临床研究有关的开支，同意禁止与本试验相关的商业、经济行为。

临床试验单位：上海市徐汇区中心医院药物临床试验机构

地址：上海市淮海中路 966 号 1 号楼 20 楼　　邮编：200031

主要研究者（签名）：签字日期：

目录

目录......3
缩略语表......5
方案摘要......6
一、研究背景......9
二、研究目的......18
三、试验药物......18
1. 药物信息......18
2. 给药方法......19
3. 药物配制......19
4. 药物管理......19
四、 试验设计......19
1. 总体设计......19
2. 研究过程......20
3. 其他限制......20
4. 评价指标......21
五、受试者管理......22
1. 入选标准......22
2. 排除标准......22
3. 脱落标准......23
4. 中止标准......23
5. 剔除标准......24
六、不良事件与评价......24
1. 不良事件的定义......24
2. 获取不良事件信息......24
3. 不良事件记录......24
4. 不良反应严重程度判定标准......24
5. 不良事件与试验药物关系的判断标准......25
6. 严重不良事件定义......25
7. 发生 SAE 时的处理......25
8. SAE 的记录与报告......25
七、合并用药......26
八、数据管理......26
1. 研究病历设计......26
2. 随机的制定......26
3. 数据录入与修改......26
4. 数据审核......27
九、统计分析......27
1. 统计分析数据库......27

2. 统计表达 27
3. 统计内容 27
4. 统计软件 28
十、研究管理 28
1. 随机盲法的实施 28
2. 质量管理 30
3. 资料填写和保存 30
4. 总结报告 30
5. 伦理学要求 31
6. 试验经费 31
7. 应急措施 31
8. 临床试验监查 31
十一、参加单位 32
1. 参加单位 32
2. 主要参加人员 32
十二、参考文献 32
附件 1.研究流程 33
附件 2.血样采集及试验其他操作的时间窗 34

缩略语表

AE	不良事件	ALB	白蛋白
ALI	急性肺损伤	ALP	碱性磷酸酶
ALT	谷氨酸丙氨酸氨基转移酶	ARDS	急性呼吸窘迫综合征
AST	谷氨酸天门冬氨酸氨基转移酶	BMI	体重指数
BUN	尿素氮	CFDA	国家食品药品监督管理总局
Cr	肌酐	CRA	监查员
CRF	病例报告表	D	天
DCF	数据澄清表	DM	数据管理员
EC	伦理委员会	GCP	药物临床试验质量管理规范
GLU	葡萄糖	GMP	产品生产质量管理规范
H	小时	Hb	血红蛋白
HBsAg	乙肝表面抗原	hCG	人绒毛膜促性腺激素
HCT	红细胞压积	HIV	艾滋病毒
IgE	免疫球蛋白 E	K^+	钾
KET	尿酮体	Kg	公斤
L%	淋巴细胞百分率	LC-MS/MS	液相色谱质谱/质谱联用
LD	半数致死量	LDH	乳酸脱氢酶
LMP	末次月经	m	米
M%	单核细胞百分率	min	分钟
ml	毫升	N%	中性粒细胞百分率
Na^+	钠	PI	主要研究者
PK	药物代谢动力学	PLT	血小板
PRO	尿蛋白	RBC	红细胞计数
rpm	每分钟转速	SAE	严重不良事件
Sepsis	脓毒症	SOP	标准操作程序
SS	安全数据集	T-BIL	总胆红素
TC	总胆固醇	TG	甘油三酯
TP	总蛋白	U	单位
UA	尿酸	UTI	乌司他丁
WBC	白细胞计数		

方案摘要

**研究药物:**注射用乌司他丁	**方案编号：**CRC-C1406
**申办单位:**广东天普生化医药股份有限公司	**适应症：**（1）急性胰腺炎；（2）慢性复发性胰腺炎；（3）急性循环衰竭的抢救辅助用药。

研究题目：一项在健康受试者中评价乌司他丁单次剂量递增的随机、双盲、安慰剂对照的安全性、耐受性研究

研究设计：单中心、随机、双盲、安慰剂平行对照、单次剂量递增

研究人群：健康成年男女

研究目的：

评价健康受试者单次静脉滴注注射用乌司他丁的安全性和耐受性。

研究药物：

乌司他丁注射剂（每支含乌司他丁 10 万单位）和安慰剂（仅含辅料）；将试验药品溶于 250 ml 的 5%葡萄糖溶液中，2h 静脉滴注。

评价指标：

安全性和耐受性：各种不良事件的发生率，包括症状或体征异常和实验室检查异常。需要重点观察的已知不良反应为过敏反应。

总体设计：

本研究采用单中心、随机、双盲、安慰剂平行对照、单次剂量递增的研究设计。

严格遵循剂量递增原则开展试验：从最低剂量开始，剂量逐步向上递增，在完成前一剂量组观察期并得出安全的评估结论后，才开展下一剂量组的试验；在剂量递增过程中出现了中止试验的标准，虽未达到预定的最大剂量，亦应中止试验；在达到最大剂量时，虽未出现终止试验的标准，亦应终止试验。

每个剂量组的临床试验结束后，须及时进行安全性分析，以便及时调整后续试验方案。

入组受试者的男女比例：女性受试者不少于 30%。

给药剂量组设置见下表：

组号	1	2	3	4	5	6	7	8	9
递增比例	起始量	100%	100%	67%	50%	50%	33%	16%	14%
用药量/万单位	30	60	120	200	300	450	600	700	800
受试者数	2+1	2+1	4+1	4+1	6+1	6+1	6+1	6+1	6+1

研究过程：

在给药前 28 天内进行筛选，选择符合研究入组标准的健康受试者。受试者在进行方案规定的各项程序前，必须获得已签署的知情同意书。具体给药方式见下：

受试者于试验前一日入住研究病房，试验期间统一清淡饮食，由研究中心提供。次日早晨 7:30 左右开始进食早餐，8:30 左右静脉滴注相应剂量的乌司他丁或安慰剂。给药前及给药开始后 2h 进行生命体征检查，给药开始后 2h 还需进行 12 导联心电图检查，受试者需在病房连续观察至给药结束后 24h（试验中将根据受试者的临床反应情况适当延长住院时间），完成出组检查后受试者离院。给药开始至给药结束后 4h 期间（共 6h）需记录受试者的饮水量和尿量。受试者分别在 11:30 和 17:30 左右进食午餐和晚餐，所有受试者的食物热量和内容均相似。

入选标准（以下 4 条均须符合）：

1. 健康的中国男性或女性受试者：根据筛选时的病史、体格检查、生命体征、12 导联心电图、胸片、实验室检查等检查项目确认健康状况良好；
2. 年龄：≥18 且≤45 周岁；
3. 体重：≥45 kg，且体重指数（BMI）：≥19 且≤28 kg/m^2；
4. 能够理解本试验的程序和方法，愿意严格遵守临床试验方案完成本试验，并签署知情同意书。

排除标准（符合以下 1 条即排除）：

1. 筛选前每日吸烟大于 5 支；
2. 筛选前 6 个月内有药物或酒精滥用史；
3. 有严重心血管、肺脏、肝脏、肾脏、血液、胃肠、内分泌、免疫、皮肤、神经疾病和精神疾病的既往病史或现病史；
4. 筛选前 5 年内有任何器官系统的恶性肿瘤史，不论治疗过或未治疗以及是否有证据表明存在局部复发或转移；
5. 有临床意义的心电图异常史；
6. 存在心血管、呼吸、肝脏、肾脏、胃肠道，内分泌或神经方面可能改变药物吸收、代谢或清除的紊乱的病史；
7. 筛选时的实验室检查中出现异常有临床意义的改变；
8. 有肾功能不全的病史或证据，表现为有临床意义的肌酐、尿素氮和尿素异常，或尿成分异常（如蛋白尿）；
9. 给药前 2 周内患严重疾病，或严重感染和外伤；
10. 给药前 3 个月内献血≥400 mL，或有严重的失血且失血量至少相当于 400 mL；
11. 过敏体质，有药物或食物过敏史者；或已知对试验药物/同类药物组分过敏；
12. 给药前 3 个月内参加过其他任何临床试验；

13. 给药前 2 周内使用过处方药、非处方药、中草药、食物补充剂（包括维生素）或使用过任何一种可能对安全性数据解释产生干扰的药物，或使用过任何已知对主要器官有常见毒性反应的药物或中草药；
14. 妊娠或哺乳期女性，或具有生育能力且不愿意在试验期间采取避孕方法的男性或女性；
15. 乙肝表面抗原阳性、丙肝抗体阳性、HIV 抗体阳性或梅毒抗体阳性者；
16. 研究者认为具有任何可能导致受试者不能完成本研究或给受试者带来明显风险的其他任何情况。

脱落标准：

1. 发生不良事件，不适合继续参加本临床试验；
2. 违背试验方案（含受试者依从性差）；
3. 失访（包括受试者自行退出）。

中止标准：

1. 在剂量递增过程中出现了严重不良反应（危及生命或严重影响日常生活，需要医疗干预和住院），虽未达到最大剂量，应中止试验；

2．在试验过程中有 1/2 以上受试者发生 CTC AE4.0 标准中分级标准 2 级或 2 级以上不良反应，应中止试验；

3. 方案有重大失误，难以评价药物；
4. 申办者因经费或管理原因要求中止；
5. 行政主管部门或伦理委员会因某种原因勒令中止试验。

剔除标准：

受试者入组后符合下列情况之一者应予剔除：

1. 同时应用研究方案之外的对研究药物有影响的其它药物；
2. 未按方案规定的剂量和方法用药；
3. 受试者纳入后发现不符合入组标准；
4. 无任何实验室检测数据记录者。

剔除病例的处理：研究者须在 CRF 中填写剔除原因，其 CRF 应保留备查；至少接受一次治疗，且有安全性记录者，应纳入安全性分析。剔除病例的随机号不能被替代。

统计方法：

所有统计分析将采用 SAS9.2 统计分析软件编程计算，以描述性统计为主，结果应结合专业分析。

预计研究时间：2014 年 07 月～2014 年 12 月

一、 研究背景

1、药物简介

乌司他丁（Ulinastatin, UTI）是一种从尿中分离纯化的分子量为 67000D 的酸性糖蛋白，含有 143 个氨基酸，是一种广谱的水解酶抑制剂。其结构的 N 末端为丙氨酸，C 末端为亮氨酸，第 10 位的丝氨酸和第 45 位的天冬酰胺上连有糖链；其分子骨架中含有两个 Kunitz 结构域，属自然界广泛存在的 Kunitz 型蛋白酶抑制剂。乌司他丁可以抑制多种蛋白、糖和脂类水解酶的活性，可稳定溶酶体膜、白细胞膜等膜结构，抑制炎症介质的过度释放；具有改善微循环和组织灌注等重要作用，可以有效地减少损伤因子对机体的损伤。

早在 1909 年 Beuer 和 Reich 就报道了人体尿液中存在有内源性的胰蛋白酶抑制剂。研究显示，当人体受到感染、发热、肿瘤、妊娠、休克、手术、给予糖皮质激素等刺激时，人体尿液中胰蛋白酶抑制剂活性会升高。进一步研究发现，在人体血浆中存在有较高浓度的蛋白酶抑制剂，当机体受到严重损伤时其被消耗，浓度明显下降，但随着机体恢复其浓度又会回升。这表明胰蛋白酶抑制剂可能具有抵抗外来刺激，减少外界损伤因子对机体的损伤，维持人体内环境平衡的作用。

由于乌司他丁是人体内源性的糖蛋白类化合物，难于对人体外源性补充的乌司他丁进行精确灵敏可靠的定量分析，故多有关乌司他丁临床药代研究的报道一直十分有限。1991 年 Jonsson 报道，瑞典 Lund Mairo 大学用 I^{125} 标记的方法进行了三例人体的药代动力学研究。1985 年日本厚生省公布的审批资料文摘中披露，持田制药曾利用放射免疫法测定 3 名健康男性静脉给药后的血药浓度，获得了乌司他丁的清除半衰期。

2、临床应用与安全性

1985 年乌司他丁首先在日本上市，用于治疗胰腺炎、急性循环障碍等疾病。1999 年国产乌司他丁在国内上市，十多年的国内临床实践表明乌司他丁对于临床急、慢性胰腺炎和休克的治疗安全有效。

截至 2012 年 12 月，乌司他丁年销售额逾 10 亿元，累计国内销售超 4100 万支（约 3.7 亿万单位），临床应用超过 300 万人次（以人均 120 万单位计）。其临床不良反应发生率低，经常记录的临床表现为：偶见白细胞减少或嗜酸粒细胞增多，偶见恶心、呕吐、腹泻，偶有 AST、ALT 上升；注射部位偶见血管痛、发红、瘙痒感、皮疹等；偶见过敏等。

2012 年底根据国家药监局的要求，广东天普公司完成并递交了首次的《乌司他丁定期安全性更新报告》（PSUR），对上市以来从各种途径收集到的不良反应做了一个汇总。自上市以来共收集到 97 例自发报告，3 例文献报告的不良事件。这些不良事件多涉及皮肤及其附件损害（29 例）、消化系统（21 例），以及以过敏样反应、寒战、发热等症状为主的全身性损害（29 例）。此外，还有少数病例涉及神经系统（6 例）、呼吸系统（3 例）、局部损害（4 例）、精神与行为障碍（4 例）、循环系统（7 例）、血液系统（5 例）、泌尿系统（3 例）、耳及乳突损害（1 例）以及其他系统（4 例）。

合计 100 例的不良事件报告中，87 例为一般不良事件（包括说明书中已载明的不良反应 74 例以及说明书中未载明的新的不良事件 13 例），这些不良事件对原患疾病影响较小，反应一般较轻微，停药即可缓解，无需特殊处理。严重不良事件 13 例（包括已知严重不良事件 7 例、新的严重的不良事件 6 例），症状包括过敏性休克（2 例）、黄疸（2 例）、血红蛋白尿（2 例）、过敏样反应（1 例）、血小板减少（2 例）、白细胞减少（1 例）、发热（1 例）以及肝功能异常（2 例）。

2013 年贵州省药监局不良反应中心承担了一个文献系统搜索评价的工作，共搜索到有效的中文文献 1588 篇，其中 129 篇对乌司他丁临床使用的安全性进行了观察，被纳入安全性研究。结果如下：

纳入研究中乌司他丁的用药人数 11232 人(含对药物不良反应 ADR 描述不清的 2 篇文献计 55 人和 1 篇健康志愿者耐受性研究 44 人)，其中最小年龄 1 岁，最大年龄不详（老年人）。

给药方式均为静脉滴注或静脉推注。

单次给药剂量最少 5 万 U，最多 120 万 U。疗程最短为单日单次，最长 14 天。

11232 名用药者中详细列举发生 ADR133 例、177 例次，另有部分患者出现血管痛、发红、瘙痒、恶心、呕吐、腹泻，但具体例次不详。损害累及 7 个系统，其中居前 3 位依次为：注射部位损害、胃肠系统损害、皮肤及附属器官损害。ADR 临床表现居前 3 位依次为：疼痛、瘙痒、皮疹和粒细胞减少。所有 ADR 临床表现中皮疹、皮肤过敏反应、瘙痒、腹痛、唾液分泌增加、粒细胞减少、寒战、高热、支气管痉挛、休克为新的 ADR（药品说明书中未列举）。具体临床表现详见表 1。

报道的 ADR 经减慢滴速、停药、对症治疗等处理后均消退或好转，未报道因 ADR 导致死亡的情形。报道支气管痉挛和休克的作者分析：ADR 病例均首次接触 UTI，均

排除其他药物的可能性，痉挛和休克均见于快速给药时。

表 1. ADR 临床表现统计表

累及系统	ADR 临床表现及例次	小计
注射部位	疼痛(22),瘙痒(20),针刺或烧灼感(5), 血管痛(4),发红(3)	54
胃肠系统	恶心(15),腹泻(10),呕吐(8),腹痛(2), 唾液分泌增加(2), 胃肠道症状(14)	51
皮肤及附属器官	皮疹(16) ,皮肤过敏反应(13),瘙痒(10)	39
血液系统	粒细胞减少(16),白细胞减少(5)	21
全身性	休克(4),寒战(1),高热(1)	6
肝胆系统	肝功能损害(2),谷草转氨酶升高(1),谷丙转氨酶升高(1)	4
呼吸系统	支气管痉挛(2)	2
合计		177

①ADR为0的研究

95篇ADR为0的研究，涉及3275名患者，涉及包括幼儿及老年人在内的各个年龄段人群，治疗范围包括急性胰腺炎、脓毒症、外科手术围术期、严重烧伤等（详见表2），单次用药剂量5～120万U，日剂量最高480万U。

表 2. ADR 为 0 的研究涉及治疗范围及人数

治疗范围	文献数量	患者数
急性胰腺炎	53	2399
肝切除术	2	63
胆管手术中	1	30
肺癌根治术中单肺通气	1	20
胃癌术后	1	69
瓣膜置换术	2	41
有机磷农药中毒	1	45
重症中暑	1	19
重型颅脑损伤并发应激性溃疡	1	90
感染性休克	2	50
小儿体外循环所致炎性反应	1	15
中重度烧伤	1	40
门脉高压脾切除	3	47
老年脓毒症	1	21
脓毒症	7	237
儿童脓毒症	1	40
外科高危病人	1	30
心肺复苏	1	19
肺挫裂伤	2	64
慢阻肺急性加重期	3	107
重症肺炎	5	149
甲型 H1N1 流感	1	23

严重烧伤	2	82
急性循环衰竭	1	25
合计	95	3275

②ADR非0的研究

35 篇 ADR 非 0 的研究（不包括健康志愿者耐受性研究），涉及 7913 名患者，主要为 16 岁以上人群，治疗范围包括急性胰腺炎、脓毒症、外科手术围术期等（详见表 3），单次用药剂量 5～40 万 U，日剂量最高 120 万 U。

表 3. ADR 非 0 的研究涉及治疗范围及人数

治疗范围	文献数	患者数
急性胰腺炎	23	1241
脓毒症	3	85
急性化脓性胆管炎	1	32
急性重症放射性肺炎	2	43
肝硬化门奇断流术围术	1	30
食管癌肝骨转移	1	1
手术麻醉期间	1	6350
循环衰竭	1	15
重症肺炎	1	30
肝切除术围术期	1	86
合计	35	7913

③特殊人群用药

有3篇文献分别报道了小儿体外循环所致炎性反应（15例，1-6岁）、儿童脓毒症（40例，平均年龄30.9月）、老年脓毒症（21例，平均年龄65.6±11.2），观察到的ADR均为0。

④ADR发生率

根据文献报道ADR例次计算乌司他丁所致ADR发生率，不同研究类型观察到的发生率不同，发生率由高到低依次为：RCT>多中心RCT>回顾性研究（详见表4）；不同治疗范围观察到的发生率也不同，发生率居前五位的由高到低依次为：循环衰竭、急性胰腺炎、肝硬化门奇断流术、急性化脓性胆管炎和重症肺炎（详见表5）。

表 4. 不同研究类型观察到的乌司他丁所致 ADR 发生率

研究类型	治疗范围	用药人数*	ADR 例数	ADR 例次	发生率（%）
回顾性	手术麻醉期间	6350	19	19	0.299
多中心 RCT1	急性胰腺炎	316	2	2	0.633
多中心 RCT2	急性胰腺炎	62	1	1	1.613
多中心 1+多中心 2	急性胰腺炎	378	3	3	0.794

本次文献荟萃 RCT	--	4812	128	148	3.076
本次文献荟萃合计	--	1116	129	148	1.326

*不包含未具体描述 ADR 例数的研究、健康志愿者耐受性研究和个案报道

表 5.　不同治疗范围观察到的乌司他丁所致 ADR 发生率（按例次）

研究类型	治疗范围	用药人数*	ADR 例数	ADR 例次	ADR 临床表现	发生率（%）
RCT	循环衰竭	15	1	2	恶心(1),呕吐(1)	13.333
RCT	急性胰腺炎	3111	101	117	疼痛(22),瘙痒(20),粒细胞减少(16),胃肠道症状(14)(恶心/呕吐/眩晕/腹泻,未分别列举具体例次),皮疹(12),恶心(11),瘙痒(10),腹泻(9),针刺或烧灼感(5),WBC 减少(5),血管痛(4),呕吐(4),发红(3),腹痛(2),肝功能损害(2),唾液分泌增加(2),休克(2),寒战(1),谷草转氨酶升高(1),谷丙转氨酶升高(1)	3.761
RCT	肝硬化门奇断流术	30	1	1	皮疹(1)	3.333
RCT	急性化脓性胆管炎	32	1	1	腹泻(1)	3.125
RCT	重症肺炎	189	3	5	恶心(3),呕吐(3),皮疹(1)	2.646
RCT	肝切除术围术期	149	1	1	高热(1)	0.671
RCT	脓毒症	322	2	2	皮疹(2)	0.621
回顾性	手术麻醉期间	6350	19	19	皮肤过敏反应(13),支气管痉挛(2),休克(4)	0.299
RCT	重症中暑	28	0	0	0	0
RCT	重型颅脑损伤并发应激性溃疡	90	0	0	0	0
RCT	有机磷农药中毒	45	0	0	0	0
RCT	严重烧伤	122	0	0	0	0
RCT	心肺复苏	19	0	0	0	0
RCT	小儿体外循环所致炎性反应	15	0	0	0	0
RCT	胃癌术后	20	0	0	0	0
RCT	外科高危病人	30	0	0	0	0
RCT	门脉高压脾切除	141	0	0	0	0
RCT	慢阻肺急性期	107	0	0	0	0
RCT	老年脓毒症	21	0	0	0	0
RCT	甲型 H1N1 流感	23	0	0	0	0
RCT	急性重症肺炎	33	0	0	0	0
RCT	急性循环衰竭	25	0	0	0	0
RCT	感染性休克	50	0	0	0	0
RCT	肺挫伤	64	0	0	0	0
RCT	肺癌根治术中单肺	20	0	0	0	0
RCT	儿童脓毒症	40	0	0	0	0
RCT	胆管手术中	30	0	0	0	0
RCT	瓣膜置换术	41	0	0	0	0
合计	--	11162	129	148	--	1.326

*不包含未具体描述 ADR 例数的研究、健康志愿者耐受性研究和个案报道

（2）在韩国 http://www.druginfo.co.kr 网站上检索到该国企业为研究乌司他丁不良反应或副作用所开展的 2 个调查研究，由于研究细节未披露，本报告书不对该 2 项研究所观察到的不良反应与乌司他丁间的因果关系进行评价及讨论：

研究一. 607 名患者为对象的调查结果：无效率为 1.81％（11/607），恶心 0.82％（5/607），呕吐、头痛各 0.66％（4/607），腹痛 0.49％（3/607），胸部不适、脐周痛、喉咙痛、呼吸困难、高胆红素血症各 0.33%（2/607），腹胀、腹水、全身衰弱、脸浮肿、胃寒、胃灼热、淀粉酶增加、胃炎、糜烂、血便、黑血便、落枕、眩晕、肺炎、口渴、低钙血症、脂肪酶增加、肝炎、贫血、急躁、睡眠障碍、瘙痒症、皮肤红疹、腰痛、甲状腺增生、尿频等各 1 例（0.16％）。

研究二. 602 名患者为对象的调查结果：无效率 10.47％（63/602），排尿困难、恶心、呕吐、神经紧张和咳嗽各 1 例（0.17％）。

上市以来，天普公司生产并销售的乌司他丁数据，请参见表 6。

表 6. 乌司他丁销售数据（截止 2012 年 12 月）

年份	销售量（支）		年份	销售量（支）	
	注射用乌司他丁（5 万单位）	注射用乌司他丁（10 万单位）		注射用乌司他丁（5 万单位）	注射用乌司他丁（10 万单位）
1999	5,470	3,940	2006	369,771	1,345,339
2000	51201	44,925	2007	690,896	1,791,091
2001	170,386	236,939	2008	995,895	2,390,712
2002	283,233	558,738	2009	1,204,950	3,547,964
2003	342,395	836,874	2010	1,620,251	4,760,528
2004	364,980	950,004	2011	1,886,507	6,330,966
2005	419,163	1,233,117	2012	1,901,104	7,644,704
合计销售量（5 万单位）：10,306,202 支<br>（10 万单位）：31,675,841 支					

以人均用药量 120 万单位/人计算，具体用药人数估算数据，请参见表 7：

表 7.乌司他丁报告期内用药人数估算数据（截止 2012 年 12 月）

批准文号	规格	报告期内销售量（支）	人均用药量	估算的用药人数（人）

国药准字 H19990133	注射用乌司他丁（5 万单位）	10,306,202	120 万单位/人，即 24 支/人	429,425
国药准字 H19990134	注射用乌司他丁（10 万单位）	31,675,841	120 万单位/人，即 12 支/人	2,639,653
合计用药人数：3,069,078				

作为一种可通过尿液排泄的人体内源性糖蛋白，乌司他丁的安全性较好。动物实验表明，乌司他丁静脉注射的急性毒性 LD50＞375 万单位/kg，相当于大于 1800 万单位/60kg/人；长期毒性试验（犬）的无毒剂量为 30 万单位/kg，相当于 972 万单位/60kg/人。乌司他丁的安全性已在十多年的临床实践中得到了证实。

3、超大剂量应用安全性研究的必要性

注射用乌司他丁（天普洛安）自 1999 年 6 月获得国家药监局批准，作为日本原研乌司他丁（Miraclid）在中国的首仿药物上市以来，注射用乌司他丁（天普洛安）的产品说明书在中国大陆沿用的是日本原研的说明书。最近一次说明书的核准日期为：2011 年 03 月 31 日。就连安全性信息，也仍旧沿用的是日本原研上市前后的数据。但日本原研的乌司他丁（商品名 Miraclid）从未进入中国市场。

批准上市以来已经发表的临床研究的文献表明，在临床一线急危重症抢救中，如休克、肺损伤、烧伤等应用乌司他丁已经远远超过上市时批准的 30 万 U 的日剂量，有高达数百万的日剂量应用（参见表 8）。针对这样远超说明书剂量的临床用药现状，临床专家一致提出，尽管从各自的用药经验中体验到天普洛安有较可靠的安全性，但还是有必要就超大剂量应用天普洛安的安全性进行研究，为临床提供更确切的循证医学证据。

为了适应临床应用的飞速发展，也为了在急危重症患者能更好地获得及时救治，天普公司认识到有必要对大剂量应用天普洛安开展上市后再研发的研究，以弥补上市前系统研究匮乏的不足。

在 2003 年 SARS 期间，注射用乌司他丁（天普洛安）在治疗 SARS 过程中显示了可能治疗全身性炎症反应性肺损伤的前景，由此，天普公司在钟南山院士的大力支持下，向药监局递交了临床研究申请，并于 2003 年 7 月获得批准用于治疗与全身性炎症反应综合症（SIRS）相关的急性肺损伤，临床研究批件号为 2003L02742，注册

分类为化学药品补充申请。

根据上述国家药监局关于对注射用乌司他丁应用于治疗肺损伤的临床研究批件相关规定，广东天普于 2005 年 9 月在安徽医大完成了一个健康受试志愿者的耐受性研究。

共 32 位受试志愿者分别接受了注射用乌司他丁（天普洛安）静脉滴注。单次 10 万、20 万、40 万、60 万、80 万、100 万和 120 万 U 的给药剂量，未观察到不良反应；在多次给药试验中，6 位受试志愿者接受了每次 30 万，每日三次（即每日 90 万）的给药剂量，持续给药 7 天，未观察到不良反应；但 6 位受试志愿者接受了每次 40 万，每日三次（即每日 120 万）的给药剂量，在第三天给药之后，有 2 位受试志愿者发生恶心、腹痛、腹泻，程度被判为轻度不良反应。研究结论为：注射用乌司他丁（天普洛安）单次给药最大安全剂量为 120 万 u/次，注射用乌司他丁（天普洛安）多次给药最大安全剂量为 90 万 u/日。

经文献检索，目前临床用于治疗肺损伤的起始剂量就达 120 万 U/日，中度至重度肺损伤剂量可能需要 240 万 U～ 400 万 U。因此，对临床安全剂量范围有必要进行进一步的探索。

据此，国家食品药品监督管理局已于 2003 年批准注射用乌司他丁进入临床试验临床试验批件号 2003L02742，注册分类为化学药品补充申请，2011 年再次在国家食品药品监督管理局备案，备案号 YPA1100512。

表 8. 大剂量乌司他丁治疗重症的文献列表

文章	作者，杂志	医院	UTI 用法	日用量	用药人数	不良反应	结局
连续性血液滤过合用乌司他丁治疗多脏器功能不全综合征	陈观生等，中国急救医学，2006,26(6):458	深圳市龙岗区人民医院肾内科	UTI 100 万 U 溶于生理盐水 20ml，缓慢静脉推注，治疗 5d	100 万 U	20 例	未报道	CVVH 结束时静脉注射 UTI，能抑制炎症因子升高，改善临床症状，促进肾功能恢复，缩短 ICU 住院天数
乌司他丁治疗慢性阻塞性肺疾病合并全身炎症反应综合征观察	王东等，医药论坛杂志	河南省驻马店市中心医院 ICU	UTI 60 万 U 静脉滴注，bid,疗程 1w	120 万 U	32 例	未报道	加用大剂量 UTI 治疗，能明显改善患者炎性反应指标和动脉血气指标，减少 MODS 的发生，降低病死率
大剂量乌司他丁与低分子肝素对脓毒症患	张栋等，中华普通外科杂志，2007:22(10):7	山西省长治医学院附属和平	UTI 20 万 U 溶于 0.9% 生理盐水 20ml,静脉推注，6	120 万 U	12 例	未报道	研究发现 UTI 与低分子肝素联合应用对于脓毒症患者而言，可能具有协同的治疗

文章	作者，杂志	医院	UTI 用法	日用量	用药人数	不良反应	结局
者的早期治疗研究	50	医院 ICU	次/d,连续使用 7d				作用，可抑制凝血系统活化
全身炎症反应综合症的靶器官保护	苏月南等，中华急诊医学杂志，2008,17(2)：187	阳江市人民医院急诊科	UTI 40 万 U，iv，q8h	120 万 U	50 例	未报道	应用 UTI 综合治疗 SIRS,与对照组相比可明显降低病死率与 MODS 发生率
乌司他丁并持续血液净化治疗多器官功能障碍综合症的研究	任宏生等，中华急诊医学杂志，2008,17(6):622	山东大学附属省立医院重症监护	UTI40 万 U+生理盐水 30ml 静脉注射，tid,连续 7 天	120 万 U	75 例	未报道	研究发现乌司他丁具有保护肝肾功能，改善血液障碍。与对照组相比，UTI 组 APACHEⅡ较对照组显著降低，器官衰竭率降低，说明乌司他丁治疗危重症疗效显著
乌司他丁对重症胰腺炎肺损伤的防治作用	张献全等，中国急救医学，2008:28(12):1060	重庆医大二附院中心 ICU	UTI30 万溶于 50ml 5%GS，微量泵入，6h/次，连续 7-14 日	120 万 U	42 例	未报道	UTI 治疗组有显著的防治胰腺炎肺损伤的作用
乌司他丁对严重脓毒症患者外周血单核细胞 Toll 样受体 4 表达的影响	刘英等，中国危重病急救医学，2009:21(9):560	泸州医学院附属医院急诊科	UTI 60 万 U+5%GS 250ml，静脉滴注，每日 2 次，连用 3d	120 万 U	40 例	未报道	UTI 对严重脓毒症患者的保护作用有可能通过抑制或水解单核细胞表面 TLR4 表达从而抑制炎症因子的释放而实现
乌司他丁辅助治疗重症脓毒症的临床疗效观察	常程等，中国临床实用医学,2010,4(12):157	北京航天总医院普外科	UTI 60 万 U+5%GS 250ml，2 次/d 静脉滴注，连续治疗 10d	120 万 U	53 例	未报道	乌司他丁用于治疗重症脓毒症具有改善临床症状、抗休克、抗炎症的效果
乌司他丁治疗老年脓毒症休克的临床研究	劳志刚等，山东医药，2011,51(41):95	广东药学院附属第一医院	UTI 40 万 U+100ml 生理盐水，静脉滴注，tid，连用 10d	120 万 U	27 例	未报道	大剂量 UTI 对器官的功能恶化起到明显的阻止作用
乌司他丁对原位肝移植术患者围术期肾功能的影响	姜一新等，中华麻醉学杂志，2011,31(8):913	昆明市第一人民医院	麻醉诱导后 1h 输注 UTI40 万 U（20ml 生理盐水），然后每 4h，重复输注 20 万 U，直至术后 48h.	首日 160 万 U，次日 120 万 U	30 例	未报道	UTI 组血清 Cr 浓度降低，肌酐清除率升高，BUN 浓度降低，提示对围术期肾功能有保护
大剂量乌司他丁对大面积烧伤患者凝血功能的影响及意义	盛志勇等，感染.炎症.修复，2005:6（3）:162	解放军总医院 304 临床部全军烧伤研究所临床部	中心静脉给予 UTI 60 万 U+100ml 0.9%生理盐水，每日 4 次，连续应用 7 天	240 万 U	4 例	未报道	UTI 显著改善大面积烧伤患者凝血功能
乌司他丁对原位肝移植术中肺氧合功能的	冯国辉，临床麻醉学杂志，2006：22(11):	北京武警总医院麻醉科	切皮后将 UTI 100 万单位加入 100ml 生理盐水中，持续	大于 200 万 U	10 例	未报道	肝移植术前和术中有明显的肺氧合功能障碍，UTI 可改善 OLT 中肺的氧合功能

文章	作者，杂志	医院	UTI 用法	日用量	用药人数	不良反应	结局
影响	821		静脉输注 1h，以后每隔 4h 重复使用，手术当天应用				
成批烧冲复合伤患者的临床救治	柴家科等，中华创伤杂志，2007:23(1):57	解放军总医院第一附属医院全军烧伤研究所	UTI 60 万 U，4 次/d	240 万 U	5 例	未报道	大剂量乌司他丁治疗后，TNF-α、IL-6 等炎症介质水平降低
创伤脓毒症患者免疫状态监测及 UTI 联合胸腺肽α1 行免疫调理的临床研究	苏磊等，解放军医学杂志，2008:33(11):1366	广州军区广州总医院 ICU	UTI 针剂 200 万 U 贝朗注射泵入（或持续泵入），每日 2 次，连续 4d 后改为 10 万 U 静脉滴入（或持续泵入），每日 2 次，再连续 6d	400 万 U /20 万 U	24 例	未报道	改善机体免疫状态，同时还改善创伤脓毒症患者的危重程度
乌司他丁对肝移植术患者凝血功能的影响	冯国辉,中华麻醉学杂志，2009,29(4):296	北京武警总医院麻醉科	UTI 首次输注量 120 万，持续 1h 输注，术中每隔 4h 重复输注，平均手术时间 12h	360-480 万	10 例	未报道	对存在凝血功能障碍的终末期肝病患者，UTI 可改善凝血功能，减少术后出血
乌司他丁与奥曲肽联合治疗重症急性胰腺炎的临床试验	张安，董晓静，中国临床药理学杂志,2008, 24(2):104	重庆医大二附院，重症监护室	乌司他丁 120 万单位+生理盐水 50ml，用微量泵入，每 6h 1 次，疗程 10 天	480 万单位	21 例	未报道	试验组和对照组的总有效率分别为 85.7% v 70.0%，痊愈率分别为 61.9% v 45.0%,有统计显著性差异。
大剂量乌司他丁治疗感染性休克的疗效分析	凌成亮、詹向阳、袁娜，实用医学杂志 2013, 29(21):3584	湖南南华大学二附院急诊科	大剂量组乌司他丁每次 100 万单位 + 50ml 生理盐水，30min 缓慢静脉推注，每小时一次，共 5 次，次日起同常规剂量组，20 万单位/次，2 次/日	大剂量组首日 500 万单位，次日起 40 万单位/日，	25 例	未报道	在 50 例 APACHE 评分为 30 的感染性休克患者中，大剂量组治疗后在血压、心率、尿量、肝功、乳酸、治疗后恢复时间、住院天数，以及病死率均显著高于常规治疗组

二、研究目的

评价健康受试者单次静脉滴注注射用乌司他丁的安全性和耐受性。

三、试验药物

1. 药物信息

注射用乌司他丁，为白色或微黄色冻干块状物或粉末，由广东天普生化医药股份

有限公司生产并提供。规格：10 万单位/瓶，有效期：36 个月，储存条件：密闭，阴凉干燥处（不超过 20℃）保存。试验药品在符合 GMP 条件的车间制备，并已按国家食品药品监督管理局审批的质量标准检验合格。

安慰剂：仅含辅料（甘露醇、氯化钠、磷酸盐缓冲液），由广东天普生化医药股份有限公司生产并提供。

2. 给药方法

将试验药品加入 250ml 5%葡萄糖溶液中，静脉滴注 2 小时。

3. 药物配制

本品溶解时，适度振摇后静置 1~2min，应注意避免剧烈振摇，以免产生大量泡沫，导致针头吸入药液时有效成分残留瓶内。

4. 药物管理

申办者提供研究药物到研究中心后，主要研究者或其授权代表签收确认，记录从申办者处收到研究药物的剂型、数量、批号及有效期等信息。研究中心一旦收到研究药物后，应当根据药物标签上的指示，进行贮存。保存地点要求安全且符合要求，只有主要研究者和其授权的研究人员可以接触到研究药物，并有专人负责管理研究药物的登记及记录。贮存在研究中心的研究药物只能按照研究方案分发。

研究者必须将研究药物的运送和分发信息准确记录在药物清点表中。监查员在中心访视期间和试验结束时，对药物清点进行监查。

每个受试者只能使用一个药物编号。研究中心接受研究药物须签署药品接收单，双人签字，一式两份，研究中心和申办单位各执一份。每份药物的使用均应在专门记录单上及时记录，双人审核签名。研究中及研究后及时收回剩余药品和已使用药品包装袋；研究结束后一并交给申办单位，双方签署药品回收单，不得把研究药物转交任何非临床试验参加者及第三方。监查员负责对研究药物的供给、使用、储藏和剩余药品的处理过程进行监查。

四、 试验设计

1. 总体设计

本研究采用单中心、随机、双盲、安慰剂平行对照、单次剂量递增的研究设计。既往文献表明，本研究药物的 AE 发生率在男女性别上没有显著差异。日本 1994 年发表的研究文章表明，羊水及新生儿尿中，乌司他丁的含量是成人尿含量的 10 倍，约

60~128U/ml，可以佐证乌司他丁对女性没有性别显著性不良反应发生率。因此，本研究入组受试者男女比例：女性不少于 30%。

严格遵循剂量递增原则开展试验：从最低剂量开始，剂量逐步向上递增，在完成前一剂量组观察期并得出安全的评估结论后，才开展下一剂量组的试验；在剂量递增过程中出现了中止试验的标准，虽未达到预定的最大剂量，亦应中止试验；在达到最大剂量时，虽未出现终止试验的标准，亦应终止试验。每个剂量组的临床评价结束后，须及时进行安全性分析，以便及时调整后续试验方案。给药剂量组设置见表 9：

表 9 单次给药剂量递增方案

组号	1	2	3	4	5	6	7	8	9
递增比例	起始量	100%	100%	67%	50%	50%	33%	16%	14%
用药量/万单位	30	60	120	200	300	450	600	700	800
受试者数	2+1	2+1	4+1	4+1	6+1	6+1	6+1	6+1	6+1

2. 研究过程

在给药前 28 天内进行筛选，选择符合研究入组标准的健康受试者。受试者在进行方案规定的各项程序前，必须获得已签署的知情同意书。具体给药方式见下：

受试者于试验前一日入住研究病房，试验期间统一清淡饮食，由研究中心提供。次日早晨 7:30 左右开始进食早餐，8:30 左右静脉滴注相应剂量的乌司他丁或安慰剂。给药前及给药开始后 30min、1h、2h（给药结束后即刻）进行生命体征检查，给药开始后 2h（给药结束后）需进行 12 导联心电图检查，受试者需在病房连续观察至给药结束后 24h（试验中将根据受试者的临床反应情况适当延长住院时间），完成出组检查后受试者离院。给药开始至给药结束后 4h 期间（共 6h）需记录受试者的饮水量和尿量。受试者分别在 11:30 和 17:30 左右进食午餐和晚餐，所有受试者的食物热量和内容均相似。

3. 其他限制

受试者在招募期间、筛选期和基线访视时，应被告知和提醒以下限制：

- 服药前 24h 到试验完成评价，不要进行高强度的体育锻炼，比如：力量训练、有氧训练、足球。
- 服药前 48h 到试验完成评价，不能饮酒。
- 服药前 48h 到试验完成评价，不能食用葡萄柚和/或葡萄柚汁，不能服用含黄嘌呤的食物或饮料，比如咖啡、巧克力、苏打水、茶、可乐、果汁。

如果有违背，必须在 CRF 的注释部分注明。

受试者及其伴侣在参加本试验前一个月及整个临床试验期间应没有育儿计划；因从受精卵形成到临床现有检测方法能够检测出怀孕之间是有一个时间过程的，这个过程的长短取决于怀孕者月经是否规律和体内激素水平升高的速度和程度，因人而异，一般受孕后 2-4 周可以测出。如果服用研究药物前受试者已受孕，在一定的时间期限内采用目前临床现有检测方法是有可能检测不出来的。研究期间及试验结束后 3 个月内，受试者及其伴侣需要采取可靠的避孕措施（输卵管结扎术、子宫切除术、禁欲以及在使用宫内避孕器或口服避孕药的情况下同时使用避孕套和/或阴道隔膜等避孕工具），否则该受试者不能参加本试验。但怀孕是受试者的个人行为，在研究者已经尽到告知义务的情况下，如果受试者或其伴侣在本试验期间或试验结束后 3 个月内仍意外怀孕，受试者将自行承担所有责任，研究者和申办者均没有赔偿的义务和责任；但受试者需立即致电研究者，研究者会根据研究药物以往的研究资料，告知受试者该研究药物的生殖毒性如何，是否继续妊娠由受试者自己决定，研究者随访其至妊娠结束。

4. 评价指标

评估受试者在临床研究期间发生的任何不良事件，包括临床表现异常和实验室检查异常，记录不良事件的临床特征、严重程度、发生时间、持续时间、处理方法及预后，判定与试验药物之间的相关性。在最后一次采血结束后需进行出组前全面体检，各项检查指标如果正常或异常但无临床意义，则只需在最后一次给药结束后 7-10 日内进行电话随访；如发现异常且有临床意义的改变，则需对该异常情况进行追踪随访，直至恢复正常或研究者认为无临床意义为止。

需要重点观测的不良反应：根据临床前研究资料，以及对上市后应用的相关文献不良反应文献报道，发现本类药主要不良反应以注射部位疼痛、瘙痒、针刺感、烧灼感、血管痛、注射部位发红、恶心、腹泻、呕吐、腹痛、皮疹、皮肤过敏反应、粒细胞减少、白细胞减少等，少见休克、寒战、高热、肝功能损害等，极少见支气管痉挛的不良反应，极少数过敏体质患者见过敏性休克的不良反应。**如试验过程中出现支气管痉挛、过敏、休克、高热等，应立即按照临床相应的抢救方法进行治疗。**药监局在 2003L02742 的临床批件中也指出：“本品为蛋白质类物质，可能有过敏的问题，在临床研究中应注意观察本品的过敏反应。”

<u>筛选期检查</u>：包括人口学资料、体格检查、病史、月经和生育史（限女性、记录 LMP）、体重、身高、生命体征（呼吸、脉率、坐位血压、口温）、血常规、尿常规、粪常规、

大便潜血试验、血生化、12 导联心电图、胸片、乙肝表面抗原（HBsAg）、丙肝抗体、HIV 抗体、梅毒抗体、血 hCG（限女性）、血清总 IgE。

试验过程中的检查：包括生命体征和 12 导联心电图检查，具体参见流程图（附件 1 和附件 2）。

出组检查：包括体格检查、生命体征、血常规、尿常规、粪常规、大便潜血试验、血生化、12 导联心电图、尿 hCG（限女性，必要时可考虑血 hCG）。

筛选期的各项检查结果 28 日内有效，超过 28 日尚未开始给药则需重新检查；除了上述规定的安全性检查外，在试验过程中，研究者如认为有必要，可加测各项检查，但需在 CRF 中记录检查理由和结果。

实验室检查主要包括以下项目：

血常规：包括白细胞总数(WBC)、红细胞计数(RBC)、血红蛋白(Hb)、红细胞压积(HCT)、血小板计数(PLT)、中性粒细胞百分率(N%)、淋巴细胞百分率(L%)、单核细胞百分率(M%)。

血生化：包括谷草转氨酶(AST)、谷丙转氨酶(ALT)、碱性磷酸酶(ALP)、乳酸脱氢酶(LDH)、总胆红素(TBIL)、总蛋白(TP)、白蛋白(ALB)、甘油三酯(TG)、胆固醇(TC)、钠(Na^+)、钾(K^+)、氯(Cl^-)、葡萄糖(GLU)、尿素氮(BUN)、肌酐(Cr)、尿酸（UA）。

尿常规：包括尿蛋白（PRO）、尿葡萄糖（GLU）、尿白细胞（WBC）、尿酮体（KET）、尿红细胞（RBC）。

粪常规及隐血：包括隐血试验、便白细胞（WBC）、便红细胞（RBC）。

五、受试者管理

1. 入选标准

以下 4 条均须符合：

1) 健康的中国男性或女性受试者：根据筛选时的病史、体格检查、生命体征、12 导联心电图、胸片、实验室检查等检查项目确认健康状况良好；
2) 年龄：≥18 且≤45 周岁；
3) 体重：≥45 kg，且体重指数（BMI）：≥19 且≤28kg/m²；
4) 能够理解本试验的程序和方法，愿意严格遵守临床试验方案完成本试验，并签署知情同意书。

2. 排除标准

符合以下 1 条即排除：

1) 筛选前每日吸烟大于 5 支；
2) 筛选前 6 个月内有药物或酒精滥用史；
3) 有严重心血管、肺脏、肝脏、肾脏、血液、胃肠、内分泌、免疫、皮肤、神经疾病和精神疾病的既往病史或现病史；
4) 筛选前 5 年内有任何器官系统的恶性肿瘤史，不论治疗过或未治疗以及是否有证据表明存在局部复发或转移；
5) 有临床意义的心电图异常史；
6) 存在心血管、呼吸、肝脏、肾脏、胃肠道，内分泌或神经方面可能改变药物吸收、代谢或清除的紊乱的病史；
7) 筛选时的实验室检查中出现异常有临床意义的改变；
8) 有肾功能不全的病史或证据，表现为有临床意义的肌酐、尿素氮和尿素异常，或尿成分异常（如蛋白尿）；
9) 给药前 2 周内患严重疾病，或严重感染和外伤；
10) 给药前 3 个月内献血≥400 mL，或有严重的失血且失血量至少相当于 400 mL；
11) 过敏体质，有药物或食物过敏史者；或已知对试验药物/同类药物组分过敏；
12) 给药前 3 个月内参加过其他任何临床试验；
13) 给药前 2 周内使用过处方药、非处方药、中草药、食物补充剂（包括维生素）或使用过任何一种可能对安全性数据解释产生干扰的药物，或使用过任何已知对主要器官有常见毒性反应的药物或中草药；
14) 妊娠或哺乳期女性，或具有生育能力且不愿意在试验期间采取避孕方法的男性或女性；
15) 乙肝表面抗原阳性、丙肝抗体阳性、HIV 抗体阳性或梅毒抗体阳性者；
16) 研究者认为具有任何可能导致受试者不能完成本研究或给受试者带来明显风险的其他任何情况。

3. 脱落标准

1) 发生不良事件，不适合继续参加本临床试验；
2) 违背试验方案（含受试者依从性差）；
3) 失访（包括受试者自行退出）。

4. 中止标准

1) 在剂量递增过程中出现了严重不良反应（危及生命或严重影响日常生活，需要医疗干预和住院），虽未达到最大剂量，应中止试验；
2) 在试验过程中有 1/2 以上受试者发生 CTC AE4.0 标准中分级标准 2 级或 2 级以上不良反应，应中止试验；
3) 方案有重大失误，难以评价药物；
4) 申办者因经费或管理原因要求中止；
5) 行政主管部门或伦理委员会因某种原因勒令中止试验。

5. 剔除标准

受试者入组后符合下列情况之一者应予剔除：
1) 同时应用研究方案之外的对研究药物有影响的其它药物；
2) 未按方案规定的剂量和方法用药；
3) 受试者纳入后发现不符合入组标准；
4) 无任何实验室检测数据记录者。

剔除病例的处理：研究者须在 CRF 中填写剔除原因，其 CRF 应保留备查；至少接受一次治疗，且有安全性记录者，应纳入安全性分析。剔除病例的随机号不能被替代。

六、不良事件与评价

1. 不良事件的定义

不良事件（Adverse Event,AE）是指自受试者签署知情同意书并入选试验开始至试验结束，发生的任何不利的事件，无论与试验药物是否有因果关系。

2. 获取不良事件信息

研究者应用简洁的语言报告直接观察到的或者受试者自发报告的所有不良事件。另外，试验开始后需定期向受试者询问不良事件情况。

3. 不良事件记录

试验期间应如实填写不良事件记录表，包括不良事件的发生时间、严重程度、持续时间、采取的措施和转归。不良事件应记录在指定的病例报告表的不良事件表中。

4. 不良反应严重程度判定标准

在填写病例报告表的不良事件表时，研究者根据 CTCAE4.0 描述不良事件的严重程度。

注意区别重度不良事件和 SAE。重度用来描述严重程度，不一定是 SAE。例如头痛可能在强度上表现为重度，但不能列入 SAE，除非它符合 SAE 标准。

5. 不良事件与试验药物关系的判断标准

根据药物与不良事件因果关系判断标准，将不良事件与受试药物应用的相关性分为五级，即肯定有关、很可能有关、可能有关、可能无关、肯定无关。并将肯定有关、很可能有关、可能有关均列为药物不良反应。将药物不良反应病例数总和作为分子，全部可供不良反应评价的入选病例作为分母，计算不良反应发生率。具体判断标准见表 5。

表 5　不良事件与试验药物关系判断标准

指标	肯定有关	很可能有关	可能有关	可能无关	肯定无关
合理的时间顺序	是	是	是	是	否
属已知受试药的反应类	是	是	是	否	否
停用受试药可改善	是	是	是或否	是或否	否
再用受试药可重复出现	是	?	?	?	否
反应可有另外解释	否	否	是	是	是

说明：？表示医德所限，不允许再重复给药；“可能无关”表示需进一步观察再作评定。

6. 严重不良事件定义

严重不良事件（Serious AdvereEvent,SAE）是指临床试验过程中发生需住院治疗、延长住院时间、导致持续或严重残疾或功能丧失、危及生命或死亡、导致先天畸形或出生缺陷、研究者认为可能严重危害受试者以及可能需要药物或外科手术防止上述结局出现的重要医学事件。

7. 发生 SAE 时的处理

若发生严重不良事件，研究医师将及时采取相应的治疗措施，确保受试者的安全。若发生与试验用药物无关的严重不良事件，受试者所需采取的医疗措施不会影响试验药物的疗效、安全性评价，其也能按期随访，可考虑继续参加。

8. SAE 的记录与报告

在临床试验过程中如发生严重不良事件，研究者应立即对受试者采取适当的救治措施并记录于 CRF 中，同时与申办方/CRO 密切配合，于 24 小时内报告试验负责单位主要研究者、伦理委员会、国家/地方食品药品监督管理局，必要时报告当地卫生行

政主管部门。研究者须认真逐项填写严重不良事件报告表，并在报告上签名及注明日期。严重不良事件报告途径：

姓名	单位	电话	传真
王燕燕	上海市徐汇区中心医院伦理委	021-54030254	021-54043676
戎练晨	广东天普生化医药股份有限公	021-60938228-33	021-60938218
国家食品药品监督管理局药品注册司研究监督处			010-88363228
上海市食品药品监督管理局药品注册处			021-63558611

七、合并用药

在研究期间，除了研究药物和用于治疗不良事件的药物，原则上禁用任何其他药品，包括处方药、非处方药、中草药或食物补充剂，如维生素。受试者如果在试验期间使用了非研究药物或者合并药物的剂量发生改变，须通知研究者。研究者须将受试者在随机化后使用的所有非研究药物和重要的非药物治疗（包括物理治疗和输血）详细记录在 CRF 中。

八、数据管理

1. 研究病历设计

根据方案要求设计病例报告表(CRF)，定义研究流程、Data Specification 及其收集的数据项，同时形成相应的 CRF 填写指南。经审查和批准后定稿，其过程要有版本管理。

2. 随机的制定

随机数字表由统计专职人员采用 SAS 9.2 版统计软件模拟产生，按各个剂量组分层区组编制随机号码，由与试验无关人员完成密封随机信封的制作。

3. 数据录入与修改

数据录入与管理由上海医药临床研究中心数据管理员负责。采用数据管理软件建立专用数据库，进行数据录入与管理。为保证数据的准确性，由两个数据管理员独立进行双份录入并校对。

对病例报告表中存在的疑问，数据管理员将产生 Data clarification form（DCF），并通过临床监查员向研究者发出询问，研究者应根据原始记录核实数据、及时回复，数据管理员根据研究者的回复，进行数据的确认与录入。

若 CRF 送至数据管理处后，研究者主动发现 CRF 上有某些错误，需要做修改。

此时研究者可通过发送“数据修改声明”(Data Correction Notification)对 CRF 数据进行修改。DM 将根据此修改数据库。

4. 数据审核

对两人独立完成任务的数据库进行逐项核查，对报告不一致的结果值，逐项核对原始记录表，予以更正，再随机抽取一定数量的 CRF 表与数据库中数据进行人工比较，以确保数据库的数据与原始病历中数据一致。

研究数据全部锁定后，由主要研究者（PI）、统计人员和申办方共同讨论统计分析计划，锁定后，对数据库的任何修改，需由临床研究负责人、生物统计学家和数据管理员共同达成书面同意方可进行。

试验结束，根据需要将 CRF 存档。数据管理中心将保存电子化数据至研究结束后 5 年，在此期间，如需稽查，数据管理中心在预约后可随时在系统上调出数据。

九、统计分析

统计分析由统计人员完成。

1. 统计分析数据库

安全数据集（SS）：至少接受一次治疗，且有安全性指标记录的实际数据。安全性缺失值不得结转；纳入可作评价的部分剔除病例，如年龄超过纳入标准的病例，但不包括使用禁用药物导致无法作安全性判断的病例。不良反应的发生率以安全集的病例数作为分母。

2. 统计表达

- 由于受试人数较少，以描述性统计为主，结果应结合专业分析。
- 定性指标以频数表，百分率或构成比描述；定量指标以均数，中位数，标准差，最大值、最小值描述。
- 报告主要采用表格表示，表格具有自明性，即具有表题，表注，例数。
- 必要时使用图形和表格结合方式的表达结果，特别是重复测量资料，以增加可读性。

3. 统计内容

病例分布

- 统计受试者分布情况，计算入组病例数、脱落例数、剔除例数及完成例数，并计算脱落率、剔除率。对入组、脱落、剔除、完成例数还按照剂量组分组描述并详细列表描述。

人口学和基线特征分析

- 对受试对象的人口学特征（年龄、性别、民族、种族、身高和体重等）和基线情况进行统计描述，其中受试者年龄（岁）=（入组日期-出生日期）/365.25。

依从性分析

- 用药依从性分析：分析是否按时按量使用试验药物，未用方案中禁用的药物和食物。
- 合并用药分析：需对各组合并用药总人数和百分比进行描述，并详细列表。且对合并用药进行编码并根据编码分剂量组进行频数表分类总结

安全性分析

安全性分析包含至少接受一次治疗，且有安全性指标记录的实际数据。安全性缺失值不得结转；包括可作评价的部分剔除病例，如年龄超过纳入标准的病例，但不包括使用禁用药物导致无法作安全性判断的病例。

- 计算不良事件和不良反应的发生率。
- 如有必要，可分系统列出不良反应发生的频率和频数，计算百分比。
- 各种严重不良事件病例的详细列表。
- 各种不良事件病例的详细列表。
- 各种不良反应病例的详细列表。
- 与试验治疗相关的不良事件以及导致停药的不良事件进行单独分析。
- 实验室指标、心电图在试验后“正常转异常”或“异常加剧”的例数和转异率。
- 列出实验室指标、心电图、体检异常病例和临床解释。
- 服药后各时间点生命体征、实验室检查、心电图、体检历时性变化情况。

4. 统计软件

采用 SAS 9.2 软件分析。

十、研究管理

1. 随机盲法的实施

1.1. 药物准备

本品为冻干粉制剂，申办方必须采取措施确保未溶解的试验药和安慰剂在外观和形状上没有区别。药物标签上印制“乌司他丁临床研究用药”，以及剂量、用法、批号、生产厂家、生产日期、有效期等。由于安慰剂不含有蛋白质，在药物溶解过程中不产

生泡沫，易破盲，因此，本研究需要通过涉盲药师配置药物实施盲法。申办方提供的试验药品（商业包装）和安慰剂不做双盲包装，但中盒（每盒十瓶）外贴有“研究用样品”的不干胶标签。

1.2. 随机入组与盲法实施

由统计编盲人员根据每个剂量组的人数，分组编制随机表，并制作密封的随机信封，每个随机号码信封上印有随机号，格式为：随机号（例如：第一组第一位受试者为：101）；随机信封内含不干胶标贴（三联），标贴内容如下：

此联贴在药品配制清单上<br><br>**在健康受试者中评价乌司他丁单次剂量递增的随机、双盲、安慰剂对照的安全性、耐受性研究**（CRC-C1406）<br>随机号;<br>研究用品：注射用乌司他丁<br>剂量组:<br>批号:<br>有效期：36 个月	此联留待研究完成后，贴在原始病例记录表CRF随机分组页<br>**在健康受试者中评价乌司他丁单次剂量递增的随机、双盲、安慰剂对照的安全性、耐受性研究**（CRC-C1406）<br>随机号;<br>研究用品：注射用乌司他丁<br>剂量组批号:<br>有效期：36 个月	此联贴在药品输液包装单上<br><br>**在健康受试者中评价乌司他丁单次剂量递增的随机、双盲、安慰剂对照的安全性、耐受性研究**（CRC-C1406）<br><br>随机号:<br>配制时间:<br>有效期:<br>注意事项:

在制作随机分配信封的同时，编盲人员也将制作一套印有随机号码的应急信封，信封内只装有随机号码相对应的研究用药分配，整套应急信封由研究员保管，以备万一发生严重不良事件（CTCAE 4.0 分级三级以上）时，可以打开应急信封（破盲）以抢救受试者，破盲一旦发生，研究员需要立即通知申办方、伦理委员会等相关方。

研究盲底由编盲人员密封保存，直至研究结束。收到申办方人员关于研究结束的通知，编盲人员将密封的盲底交给申办方。本研究不需揭盲，研究完成后，收集全部的原始病例记录表，由不参与研究的涉盲药师（或护士）将三联标签中的最后一联标签贴到原始病例记录表的随机入组栏内。

盲法给药：本耐受性研究需要专门涉盲药师（或护士）的协助。

每一个筛选合格的受试者入住之后，研究员（或研究员授权的研究助理）将按照顺序分配给受试者一个随机号码，涉盲药师（或护士）将根据受试者的随机号码，打开印有对应随机号码的密封信封，根据随机信封内指定的药物分配，取相应研究用药（天普洛安，或安慰剂），每一瓶注入 2ml 5%葡萄糖水溶解，适度振摇（请避免剧烈振摇）后，抽取溶解后的水溶液注入滴注的容器。配制后的容器需要贴上述第三联标签。

2. 质量管理

临床监查：由申办方的临床研究监查员，定期进行试验医院现场监查访问，以保证临床试验方案的所有内容都得到严格遵守，并对原始资料进行检查，以确保与 CRF 上的内容一致。

培训：临床试验开始前，主要研究者应对参与试验的研究人员进行试验方案的培训。熟读和了解本临床试验方案的内容，掌握 GCP 原则，统一记录方式与判断标准，严格按照方案执行。

规范操作：申办单位遵守药物临床试验质量管理规范和药物注册管理办法规定，在符合 GMP 条件的车间制备研究药物，并接受严格的质量检查。实验室检查由研究中心按照标准操作程序(SOP)进行检测。研究中心临床检验室须按规定进行室内质控，并获得卫生部临床检验中心质量评价合格证书。

数据核实：确保临床试验中各项结论来源于原始数据。在临床试验和数据处理阶段均有相应的数据管理措施，以保证数据的可靠性。

3. 资料填写和保存

病例报告表（CRF），由研究者在临床观察过程中逐项填写，CRF 需由研究中心主要研究者核实后签名。原始检验报告单均应附在研究病历中，研究病历逐项填写后，需负责医师签名。

在本临床试验期间，研究人员应保存所有原始资料（包括实验室检查报告）。临床试验各种原始数据应及时、真实、准确、完整记录，化验单应保留。临床试验资料应保存至临床试验终止后 5 年，申办者应保存临床试验资料至试验药品被批准上市后 5 年。

4. 总结报告

达到总病例数要求并进行核查后，数据统计中心按照药物临床试验统计学指导原则要求进行数据分析，研究中心根据统计分析报告，按 GCP 和药物临床试验指导原

则的要求写出临床试验总结。

5. 伦理学要求

伦理委员会：临床试验开始前，临床试验方案及知情同意书等相关资料需由伦理委员会审议同意并签署批准意见后方可实施。在临床试验进行期间，临床试验方案的重大修改，应经伦理委员会批准后方可执行。

受试者权益保障：临床研究者必须向受试者说明参加临床试验是自愿的，而且在试验的任何阶段有权随时退出试验而不会遭到歧视，其医疗待遇和权益不受任何影响，仍可继续得到其它有效治疗。必须使受试者了解到参加试验及在试验中的个人资料均属保密。还需告知受试者临床试验的性质、试验的目的、预期可能的受益和可能发生的风险和不便，告知受试者可能被分配到试验的不同组别、可供选用的其他治疗方法以及符合《赫尔辛基宣言》规定的受试者的权利和义务等，使受试者有充分的时间考虑是否参加试验，并签署知情同意书。

文件备案：将经研究中心医学伦理委员会批准的方案、病例报告表、知情同意书等文件上报国家食品药品监督管理局备案。

6. 试验经费

本试验全部经费均由申办单位提供，另签署合同说明具体经费提供的情况。

7. 应急措施

注射用乌司他丁的不良反应偶见白细胞减少或嗜酸粒细胞增多、恶心、呕吐、腹泻、过敏反应；注射部位偶见血管痛、发红、瘙痒感、皮疹等。其中，过敏性休克为重大的不良反应，若发生过敏性休克，应立即中止给药，并按照相关标准操作规程进行处理。

试验场所必须配备必要的医疗抢救设备、急救药品。组成应急小组，试验期间密切观察受试者的不良反应。

8. 临床试验监查

申办者任命具备适当的医学、药学或相关专业学历，并经过必要的训练，熟悉GCP 和有关法规的人员作为本临床试验的监查员，以监查和报告试验的进行情况和核实数据，保证临床试验中受试者的权益受到保障，试验记录与报告的数据准确、完整无误，保证试验遵循已批准的方案、GCP 和有关法规。

通过对临床试验中不良事件和严重不良事件进行监查，以保证所有的不良事件均得到准确、可靠、迅速的记录和报告。

十一、参加单位

1. 参加单位

单位名称	在试验中的性质
广东天普生化医药股份有限公司	申办单位
上海市徐汇区中心医院药物临床试验机构	临床研究单位
上海医药临床研究中心有限公司	数据管理和统计分析

2. 主要参加人员

在试验中的职责	姓名	单位名称
主要研究者	余琛	上海市徐汇区中心医院药物临床试验机构
助理研究者	陈倩	上海市徐汇区中心医院药物临床试验机构
申办方项目负责人	戎练晨	广东天普生化医药股份有限公司
临床监查员（盲）	戎练晨	广东天普生化医药股份有限公司
临床监查员（非盲）	徐虹	广东天普生化医药股份有限公司
统计分析人员	付广建	上海医药临床研究中心有限公司

十二、参考文献

（1） 国家食品药品监督管理局. 药品注册管理办法. 2007.
（2） 国家食品药品监督管理局. 药物临床试验质量管理规范. 2003.
（3） 日本注射用乌司他丁说明书. 2011.
（4） 国产注射用乌司他丁说明书. 2011.

附件 1.研究流程

项目	筛选期	试验期		
访视次数	V1	V2	V3	V4
访视时间	D-28～D-1	D0	D1	D2
知情同意	√			
人口学资料	√			
病史	√			
体格检查	√	√		√
身高、体重	√			
生命体征 [1]	√	√	√	√
血常规	√			√
尿常规	√			√
粪常规及隐血	√			√
血生化	√			√
hCG[2]	√	√		√
12 导联心电图 [3]	√		√	√
胸片 [4]	√			
血清总 IgE	√			
血清病毒学检	√			
饮水量 [5]			√	
尿量 [5]			√	
入选/排除标准	√	√		
分配筛选号	√			
分配随机号		√		
给药			√	
临床观察		√	√	√
合并用药		√	√	√
不良事件		√	√	√

注释：

1. 给药前及给药开始后 30min、1h、2h(给药结束后即刻)进行生命体征检查。
2. 筛选期检查血 hCG，D0 和出组前检查尿 hCG；对于 D0 天末次月经已经接近 1 个月的女性受试者，可由研究者决定是否检测血 hCG。
3. 给药开始后 2h (给药结束后即刻)采集 12 导联心电图。
4. 亦可在 D0 完成。
5. 需记录 D1 给药开始至给药结束后 4h 内（约 6h）受试者的饮水量和尿量。

附件 2.血样采集及试验其他操作的时间窗

操作	时间点	时间窗
给药	所有给药	2h±10min
生命体征	给药前	给药前 1h 内
	给药开始后 30min	±5min
	给药开始后 1h	±10min
	给药开始后 2h（给药结束后即刻）	±15min
12 导联心电图	给药开始后 2h（给药结束后即刻）	±15min

注：生命体征和 12 导联心电图操作不分先后。
